# Intercellular communication between hepatic stellate cells and myofibroblasts mediated by osteopontin and FGF18 promotes liver fibrosis

**DOI:** 10.1016/j.isci.2025.112932

**Published:** 2025-06-17

**Authors:** Takao Seki, Sachiko Komazawa-Sakon, Takashi Nishina, Tetuo Mikami, Hideo Yagita, Katsuhide Okunishi, Minoru Tanaka, Yuichi Tsuchiya, Hiroyasu Nakano

**Affiliations:** 1 Department of Biochemistry, Faculty of Medicine, Toho University, 5-21-16 Omori-Nishi, Ota-ku, Tokyo 143-8540, Japan; 2 Unit of Host Defense, Faculty of Medicine, Toho University, 5-21-16 Omori-Nishi, Ota-ku, Tokyo 143-8540, Japan; 3 Department of Pathology, Faculty of Medicine, Toho University, 5-21-16 Omori-Nishi, Ota-ku, Tokyo 143-8540, Japan; 4 Department of Immunology, Faculty of Medicine and Graduate School of Medicine, Juntendo University, 2-1-1 Hongo, Bunkyo-Ku, Tokyo 113-8421, Japan; 5 Department of Regenerative Medicine, Research Institute, National Center for Global Health and Medicine, 1-21-1, Toyama, Shinjuku-ku, Tokyo 162-8655, Japan; 6 Laboratory of Stem Cell Regulation, Institute for Quantitative Biosciences, The University of Tokyo, 1-1-1, Yayoi, Bunkyo-ku, Tokyo 113-0032, Japan; 7 Department of Biochemistry, Faculty of Pharmaceutical Sciences, Toho University, 2-2-1 Miyama, Funabashi-shi, Chiba 274-8510, Japan; 8 Research Administration Organization, Toho University, 5-21-16 Omori-Nishi, Ota-ku, Tokyo 143-8540, Japan

**Keywords:** Cell biology, Fibrosis, Transcriptomics

## Abstract

Hepatic stellate cells (HSCs) play a central role in the development of liver fibrosis. We previously showed that fibroblast growth factor 18 (FGF18) promotes liver fibrosis by increasing HSC proliferation. However, the underlying mechanisms remain incompletely understood. Here, we showed that FGF18 efficiently induced osteopontin (*Spp1*/OPN) expression in culture-activated αSMA^+^ HSCs, but not in freshly prepared quiescent HSCs. Notably, OPN upregulated profibrotic genes only in quiescent HSCs, suggesting that the activation status of HSCs influences their responsiveness to FGF18 and OPN. Furthermore, FGF18 and TGFβ synergistically increased *Spp1/*OPN expression in culture-activated αSMA^+^ HSCs. Immunohistochemical analyses of murine liver fibrosis models revealed that OPN was expressed predominantly in αSMA^+^ myofibroblasts, but not in desmin^+^ quiescent HSCs. The cell-cell communication analyses further revealed that myofibroblast-derived *Spp1* signaled to HSCs in fibrotic livers. Together, FGF18 initiates a feedforward loop between quiescent and activated αSMA^+^ HSCs/myofibroblasts via OPN signaling, thereby driving fibrosis progression.

## Highlights

• FGF18 stimulates osteopontin (OPN) production in culture-activated αSMA^+^ HSCs
• OPN induces profibrotic gene expression in freshly isolated quiescent HSCs
• *Spp1* from myofibroblasts transmits signals to HSCs in a murine liver fibrosis model
• FGF18 initiates a feed-forward loop between quiescent HSCs and activated HSCs

## Graphical abstract

**Figure:**
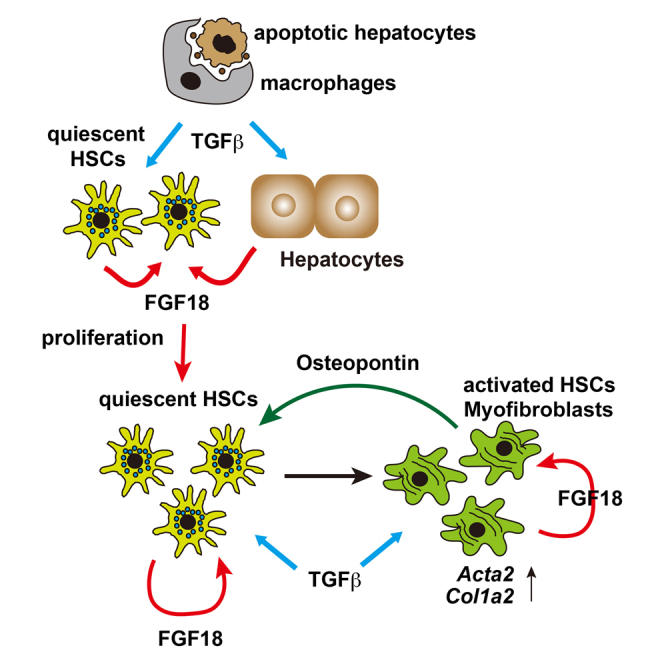

## Introduction

Liver fibrosis occurs due to chronic liver injury caused by various factors, such as viral infections, excessive alcohol consumption, lipid accumulation, and autoimmune diseases.^1^^,^^2^ Over time, liver fibrosis can progress to liver failure, cirrhosis, and cancer, making it a major contributor to cause-specific mortality worldwide.^3^^,^^4^ Notably, metabolic dysfunction-associated steatohepatitis (MASH) has recently emerged as a leading cause of liver fibrosis. Both simple steatosis and MASH are types of metabolic dysfunction-associated steatotic liver disease (MASLD).^5^^,^^6^ Approximately 10% of MASLD patients develop MASH, which is characterized by hepatocyte steatosis, inflammation, and cell death.^5^^,^^6^ While lipid-mediated toxicity and oxidative stress are believed to drive hepatocyte death in MASH, the detailed mechanisms remain incompletely understood.^7^^,^^8^

Hepatic stellate cells (HSCs) are key mediators of liver fibrosis.^9^^,^^10^ In their quiescent state, these cells contain retinol-storing lipid droplets and express characteristic markers, such as *lecithin retinol acyltransferase* (*Lrat*) and *desmin* (*Des*).^11^ Upon activation, HSCs lose *Lrat* expression and transition into αSMA-positive myofibroblasts. These myofibroblasts produce large amounts of extracellular matrix (ECM) and express high levels of profibrotic genes.^9^^,^^10^ Various cytokines, such as Hedgehog, connective tissue growth factor (CTGF), and transforming growth factor β (TGFβ),^12^ drive HSC activation. A recent study highlighted the critical role of autocrine signaling circuits involving neurotrophin 3 and its receptor in liver fibrosis.^10^ However, whether additional factors mediate intercellular communication between quiescent HSCs and activated HSCs/myofibroblasts during fibrosis progression remains unclear.

Fibroblast growth factors (FGFs) are a family of 22 structurally related proteins that regulate a wide range of biological functions. The extracellular FGF family is divided into the FGF1, FGF4, FGF7, FGF8, and FGF19 subfamilies, which signal through cognate receptors (FGFR1–4).^13^^,^^14^ Members of the FGF1, FGF4, FGF7, and FGF8 subfamilies act in a paracrine or autocrine manner, whereas the FGF19 subfamily functions as an endocrine hormone.^14^ FGFs promote and attenuate liver fibrosis in a context-dependent manner.^15^ For example, FGF21, a member of the FGF19 subfamily, reduces hepatic inflammation and fibrosis in patients with MASH when it is administered as an analog.^16^^,^^17^

We previously reported that hepatocyte-specific *Cflar*-deficient (*Cflar*^*LKO*^) mice are highly susceptible to TNF-induced hepatocyte apoptosis.^18^ Compared with control mice, *Cflar*^*LKO*^ mice rapidly develop liver fibrosis fed a choline-deficient, ethionine-supplemented (CDE) diet, and FGF18 was identified as a novel factor that promotes liver fibrosis.^19^ FGF18 stimulates HSC proliferation and increases *cyclin D1* (*Ccnd1*) expression, while also suppressing TGFβ-induced profibrotic gene expression *in vitro*.^19^ Notably, FGF18 overexpression alone induces liver fibrosis *in vivo*,^19^ suggesting that its profibrotic effects are not limited to promoting HSC proliferation. This finding implies that FGF18 may act on additional targets to drive fibrosis progression.

Osteopontin (OPN), an ECM protein, mediates cellular signaling by binding to CD44 or integrin receptors.^20^ In the liver, OPN is expressed by various cell types, including cholangiocytes, macrophages, T cells, and HSCs.^21^ OPN is implicated in numerous pathological processes, including inflammation, tumorigenesis, and liver fibrosis.^22^ Previous studies have shown that autocrine activation of HSCs by OPN induces profibrotic gene expression, promoting liver fibrosis.^23^ Conversely, the deletion of *Spp1* (encoding OPN) has been shown to alleviate liver fibrosis in multiple murine models.^23^^,^^24^ These findings establish OPN as a critical promoter of liver fibrosis and tumorigenesis under pathological conditions. However, the signals that regulate *Spp1* expression and the role of OPN in intercellular communication among HSCs remain unclear.

In this study, we identified OPN as a novel downstream effector of FGF18 in promoting liver fibrosis. FGF18 specifically induced *Spp1* expression in culture-activated αSMA^+^ HSCs, but not in freshly isolated quiescent HSCs. OPN secreted by culture-activated αSMA^+^ HSCs selectively stimulated quiescent HSCs, leading to the upregulation of profibrotic genes. Furthermore, we showed that FGF18 and TGFβ synergistically increased *Spp1* expression in culture-activated αSMA^+^ HSCs. The immunohistochemical analyses and cell-cell communication analysis further revealed that *Spp1* derived from myofibroblasts acted on HSCs in murine fibrosis models. Together, our results suggest that FGF18 drives a feed-forward loop between quiescent and activated HSCs/myofibroblasts via OPN signaling, thereby accelerating liver fibrosis progression.

## Results

### FGF18 and TGFβ increase *Spp1* expression in freshly isolated HSCs

**Figure 1:**
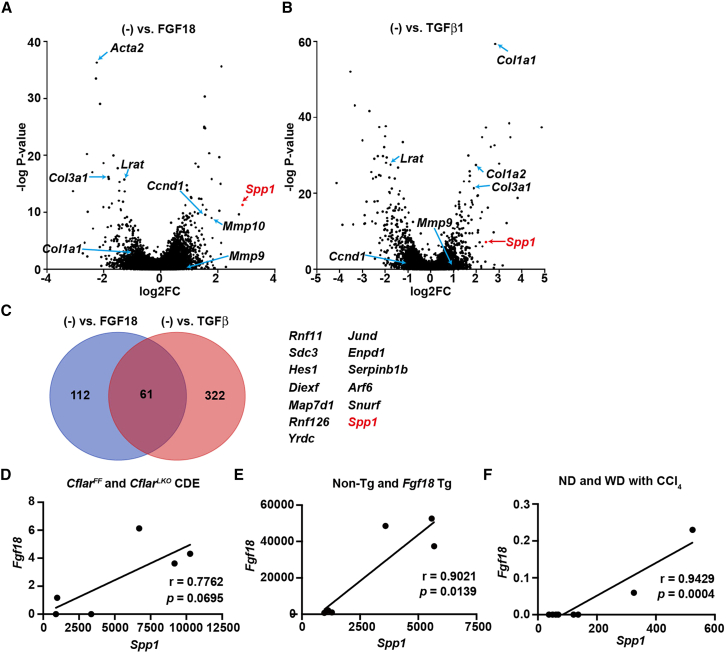
FGF18 and TGFβ increase *Spp1* expression in freshly isolated HSCs (A and B) HSCs were isolated from wild-type female mice (>24 weeks old) using Nycodenz gradient centrifugation, as described in the STAR Methods. Freshly isolated HSCs were serum-starved for 24 hours and either left untreated or stimulated with FGF18 (100 ng/mL, A) or TGFβ1 (1 ng/mL, B) for 24 hours. Gene expression profiles were assessed by RNA-seq, and results from triplicate samples are shown in volcano plots. The selected genes are indicated by arrows. (C) Venn diagram showing genes upregulated by FGF18, TGFβ1, or both. The numbers indicate genes that are uniquely or commonly upregulated. The top 13 representative genes are listed. (D–F) Correlations between *Spp1* and *Fgf18* expression in bulk RNA-seq datasets. The data were retrieved from GSE188273 (D and E) and GSE99010 (F). Relative levels of *Fgf18* and *Spp1* in whole liver samples from *Cflar*^*F/F*^ and *Cflar*^*LKO*^ mice fed a CDE diet for 4 weeks (*n* = 3 mice per group) (D), non-Tg and *Fgf18*-Tg mice (*n* = 3 mice per group) (E), or wild-type mice injected with CCl_4_ and fed either a normal diet (ND) or a Western diet (WD) for 12 or 24 weeks (pooled samples from *n* = 4 mice per condition) (F) are shown. Pearson’s correlation coefficients and two-tailed *p* values were calculated. See also Figure S1 and Table S1.

Given that FGF18 contributes to liver fibrosis by promoting HSC proliferation while mitigating TGF-β-induced profibrotic gene expression,^19^ we hypothesized that FGF18 might regulate additional target genes involved in liver fibrosis. We stimulated freshly isolated quiescent HSCs with FGF18 or TGFβ and performed bulk RNA sequencing (RNA-seq) analysis to identify these targets. Consistent with previous findings,^19^ FGF18 upregulated *Ccnd1* expression while downregulating the expression of profibrotic genes, including *collagen type 1 alpha 1 chain* (*Col1a2*), *Col3a1*, and *Acta2* (encoding αSMA)(Figure 1A; Table S1). In contrast, TGFβ stimulated the expression of *Col1a1*, *Col1a2*, and *Col3a1* but downregulated the expression of *Ccnd1* (Figure 1B). Interestingly, the expression levels of 61 genes were increased by both stimuli (Figure 1C). Given that FGF18 and TGFβ may cooperate to promote liver fibrosis under certain conditions,^19^ focusing on genes coinduced by both stimuli is reasonable. We focused on *Spp1*, which encodes OPN, as *Spp1* has been implicated in tissue repair and fibrosis.^22^^,^^25^ *Spp1* expression correlated strongly with *Fgf18* expression in multiple murine models of liver fibrosis, including *Cflar*^*LKO*^ mice with CDE diet-induced fibrosis, hepatocyte-specific *Fgf18* transgenic mice, and wild-type mice fed a Western diet and injected with CCl_4_ (Figures 1D–1F).^19^^,^^26^ Furthermore, *Spp1* expression was correlated with the expression of other profibrotic markers, such as *Col1a2*, *Col3a1*, *Acta2*, *Tgfb1*, *Tgfb2*, and *Tgfb3* (Figure S1). Collectively, these results suggest that FGF18 induces *Spp1* expression in HSCs, which is strongly correlated with the *in vivo* expression of profibrotic genes.

### FGF18 robustly induces OPN production in culture-activated αSMA^+^ HSCs

**Figure 2:**
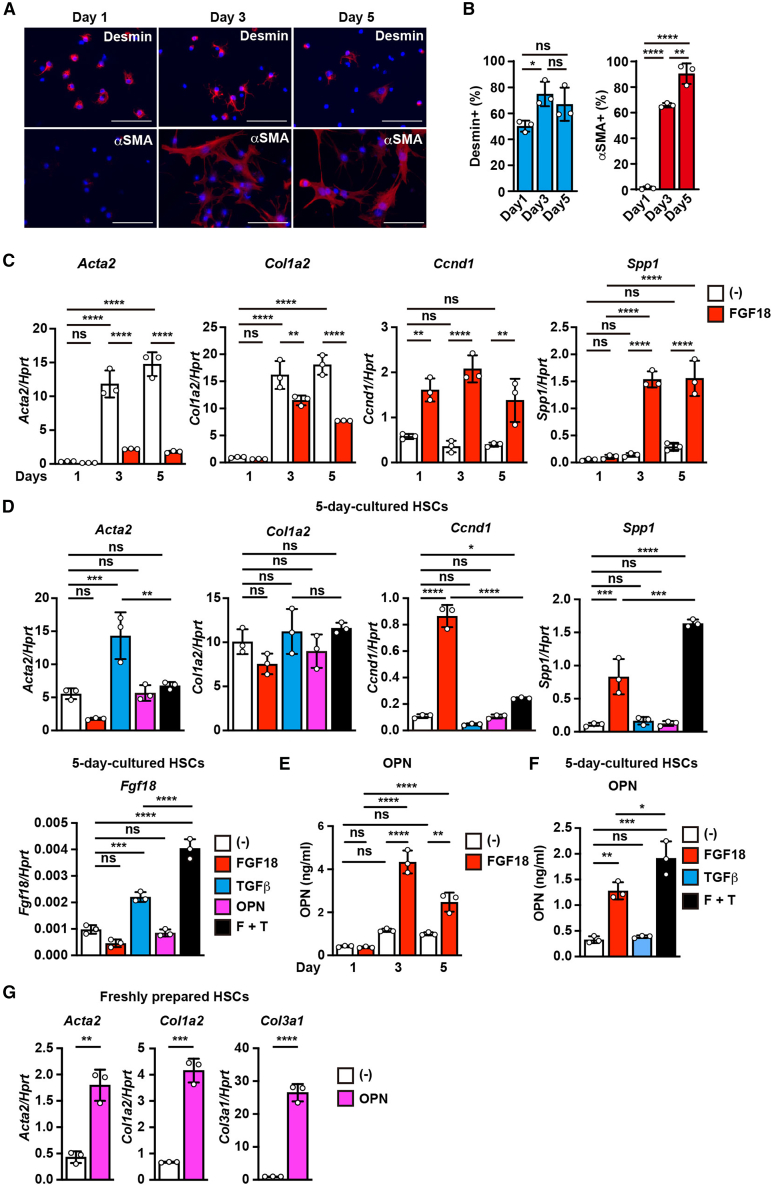
FGF18 robustly induces OPN production in culture-activated αSMA^+^ HSCs (A and B) Freshly prepared quiescent HSCs spontaneously express αSMA after 3 or 5 days of culture without any stimulation. HSCs were isolated as described in Figure 1A, plated on standard culture dishes, and serum-starved for the indicated durations without any external stimuli. The cells were stained with anti-desmin or anti-αSMA antibodies (A). Scale bars: 100 μm. The percentages of desmin^+^ and αSMA^+^ cells among DAPI-positive nuclei were quantified from three randomly selected fields (B). Results are mean ± SD of triplicate samples. (C) FGF18 robustly increases *Spp1* expression in HSCs cultured for 3 or 5 days. HSCs were isolated and serum-starved as in (A), and then left untreated or stimulated with FGF18 (100 ng/mL) for 24 h. Gene expression was analyzed by qPCR. Results are mean ± SD of triplicate samples. (D and F) FGF18 and TGFβ synergistically induce *Spp1* expression and OPN production in 5-day-cultured HSCs. Freshly prepared HSCs were serum-starved for 5 days and treated with FGF18 (100 ng/mL), TGFβ1 (1 ng/mL), OPN (100 ng/mL), or a combination of FGF18 and TGFβ1 for 24 h. Gene expression and secreted OPN protein levels were assessed by qPCR and ELISA, respectively. Results are mean ± SD of triplicates. (E) FGF18 induces OPN production in HSCs cultured for 3 or 5 days. Freshly prepared HSCs were treated as in (C), and OPN levels in the culture supernatants were measured by ELISA. Results are mean ± SD of triplicate samples. (G) Freshly prepared HSCs were serum-starved for 24 h and then left untreated or stimulated with recombinant OPN (100 ng/mL) for 24 h. Gene expression was analyzed by qPCR. Results are mean ± SD of triplicate samples. All data are representative of at least two independent experiments. Statistical significance was determined by two-way ANOVA with Tukey’s multiple comparisons (C and E), one-way ANOVA with Tukey’s (B and D) or Dunnett’s test (F), or two-tailed unpaired Student’s *t* test (G). ∗*p* < 0.05; ∗∗*p* < 0.01; ∗∗∗*p* < 0.001; ∗∗∗∗*p* < 0.0001; ns, not significant.

We first investigated whether the activation status of HSCs plays a role in *Spp1* expression. Freshly isolated quiescent HSCs become spontaneously activated after several days of culture, even in the absence of external stimuli.^27^^,^^28^ Indeed, freshly isolated quiescent HSCs expressed desmin, a marker of quiescent HSCs, but not αSMA, a marker of activated HSCs and myofibroblasts (Figures 2A and 2B). After 3–5 days in culture, most HSCs began expressing αSMA, a hallmark of activation. These cells also presented increased expression of fibrosis-related genes such as *Col1a2* and *Acta2*, although FGF18 attenuated this increase slightly (Figure 2C). These findings suggest that HSCs cultured for 3 to 5 days resemble activated HSCs or myofibroblasts *in vivo*. Interestingly, FGF18 consistently induced the expression of *Ccnd1*, a proliferation-related gene, regardless of the HSC activation status. FGF18 modestly induced *Spp1* expression in HSCs cultured for 1 day (1.9-fold higher than that in untreated controls). In contrast, *Spp1* expression was markedly increased in HSCs cultured for 3 or 5 days, with 10.9- and 5.3-fold increases, respectively (Figure 2C). These findings indicate that the robust induction of *Spp1* expression by FGF18 requires the prior activation of HSCs.

We next examined whether FGF18 and TGFβ acted cooperatively. In HSCs cultured for 5 days, FGF18 reduced TGFβ-induced *Acta2* expression, whereas TGFβ suppressed FGF18-induced *Ccnd1* expression (Figure 2D). In contrast, the combination of FGF18 and TGFβ substantially increased the expression of both *Fgf18* and *Spp1* (Figure 2D). ELISA further confirmed that FGF18 alone, and more robustly in combination with TGFβ, increased OPN production (Figures 2E and 2F). Notably, addition of OPN to 5-day-cultured HSCs did not further increase fibrosis-related gene expression, likely because of their already activated state (Figure 2D). However, OPN markedly induced the expression of these genes in freshly isolated quiescent HSCs (Figure 2G). These findings suggest that activated HSCs produce OPN, which stimulates neighboring quiescent HSCs, thereby establishing a feedforward loop that promotes liver fibrosis progression.

### FGF18 transmits signals through FGFR1 and FGFR2 in a MEK-dependent manner

**Figure 3:**
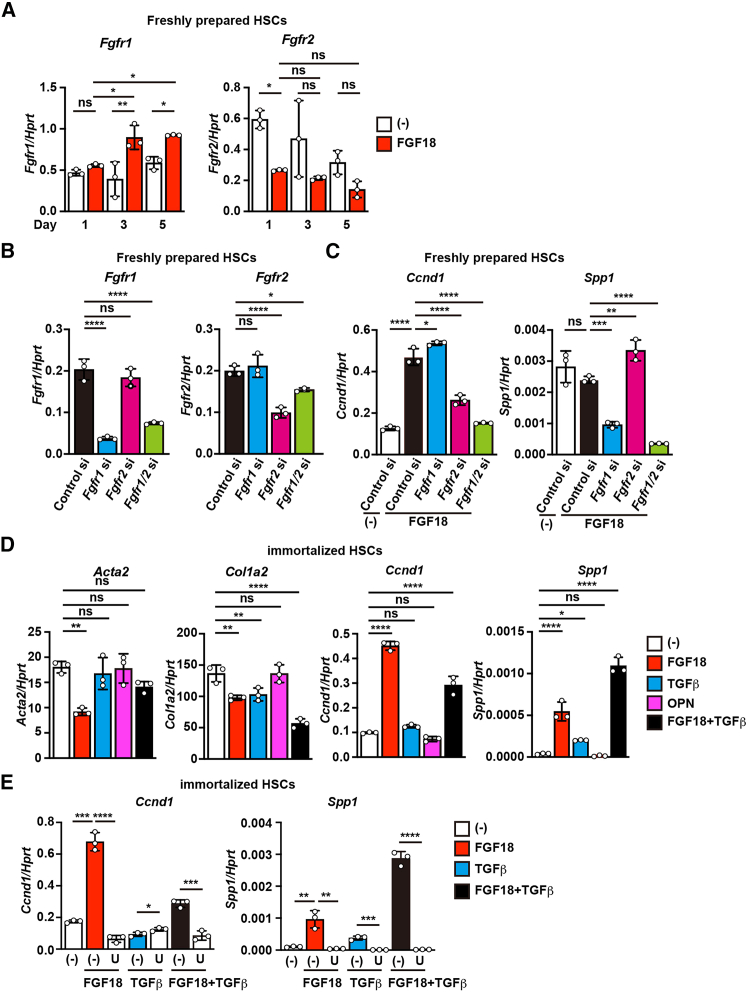
FGF18 transmits signals through FGFR1 and FGFR2 in a MEK-dependent manner (A) Freshly prepared quiescent HSCs were cultured as described in Figure 2C, and the expression of *Fgfr1* and *Fgfr2* was determined by qPCR. Results are mean ± SD of triplicate samples. (B) Freshly prepared quiescent HSCs were serum-starved for 24 h and then transfected with the indicated siRNAs, and the expression of *Fgfr1* and *Fgfr2* on day 2 after transfection was subsequently determined via qPCR. Results are mean ± SD of triplicate samples. (C) Freshly prepared quiescent HSCs were treated with the indicated siRNAs as described in (B) and then left untreated or stimulated with FGF18 (100 ng/mL) for 24 hours. The expression of *Ccnd1* and *Spp1* was determined via qPCR. Results are mean ± SD of triplicate samples. (D and E) Immortalized HSCs described in the STAR Methods were stimulated with the indicated agents in the absence (D) or presence (E) of U0126 (U) (10 μM) for 24 h, after which the expression of the indicated genes was determined by qPCR. Results are mean ± SD of triplicate samples. All results are representative of two independent experiments. Statistical significance was determined by one-way ANOVA with Dunnett’s multiple comparison test (B and D) or two-way ANOVA with Tukey’s multiple comparisons (A, C, and E). ∗*p* < 0.05; ∗∗*p* < 0.01; ∗∗∗*p* < 0.001; ∗∗∗∗*p* < 0.0001; ns, not significant.

FGFs bind to FGFR1–4 to initiate signaling pathways, leading to the activation of the MEK/ERK pathway.^29^ Our previous single-cell RNA sequencing (scRNA-seq) analysis revealed that HSCs predominantly express *Fgfr1* and *Fgfr2* but not *Fgfr3* or *Fgfr4*.^19^ We first examined the expression dynamics of *Fgfr1* and *Fgfr2* in freshly isolated HSCs before and after culture, with or without FGF18 treatment to determine which FGF receptors mediate FGF18 signaling. *Fgfr1* expression remained stable throughout the culture period, with a slight increase upon FGF18 stimulation (Figure 3A). In contrast, *Fgfr2* expression gradually decreased and was further reduced by FGF18, although not to a statistically significant extent (Figure 3A). We performed siRNA-mediated knockdown of *Fgfr1* or *Fgfr2* in freshly isolated quiescent HSCs followed by FGF18 stimulation to functionally assess receptor involvement. The knockdown of *Fgfr1* or *Fgfr2* was verified by qPCR (Figure 3B). Under these conditions, *Fgfr2* knockdown significantly suppressed FGF18-induced *Ccnd1* expression, whereas *Fgfr1* knockdown specifically reduced FGF18-induced *Spp1* expression (Figure 3C). Due to the limited number of primary HSCs available for mechanistic studies, we established immortalized HSCs using SV40 T antigen. In immortalized HSCs, FGF18-induced *Ccnd1* expression, and FGF18 plus TGFβ synergistically increased *Spp1* expression. This gene expression pattern closely recapitulates that observed in culture-activated αSMA^+^ HSCs (Figure 3D), indicating that immortalized HSCs retain key transcriptional responses to fibrogenic stimuli. Importantly, treatment with a MEK inhibitor completely abolished both the FGF18- and FGF18 plus TGFβ-induced expression of *Ccnd1* and *Spp1* (Figure 3E), highlighting the essential role of the MEK/ERK pathway in the FGF18-induced gene expression.

### OPN is localized in αSMA^+^ activated HSCs/myofibroblasts but not desmin^+^ quiescent HSCs *in vivo*

**Figure 4:**
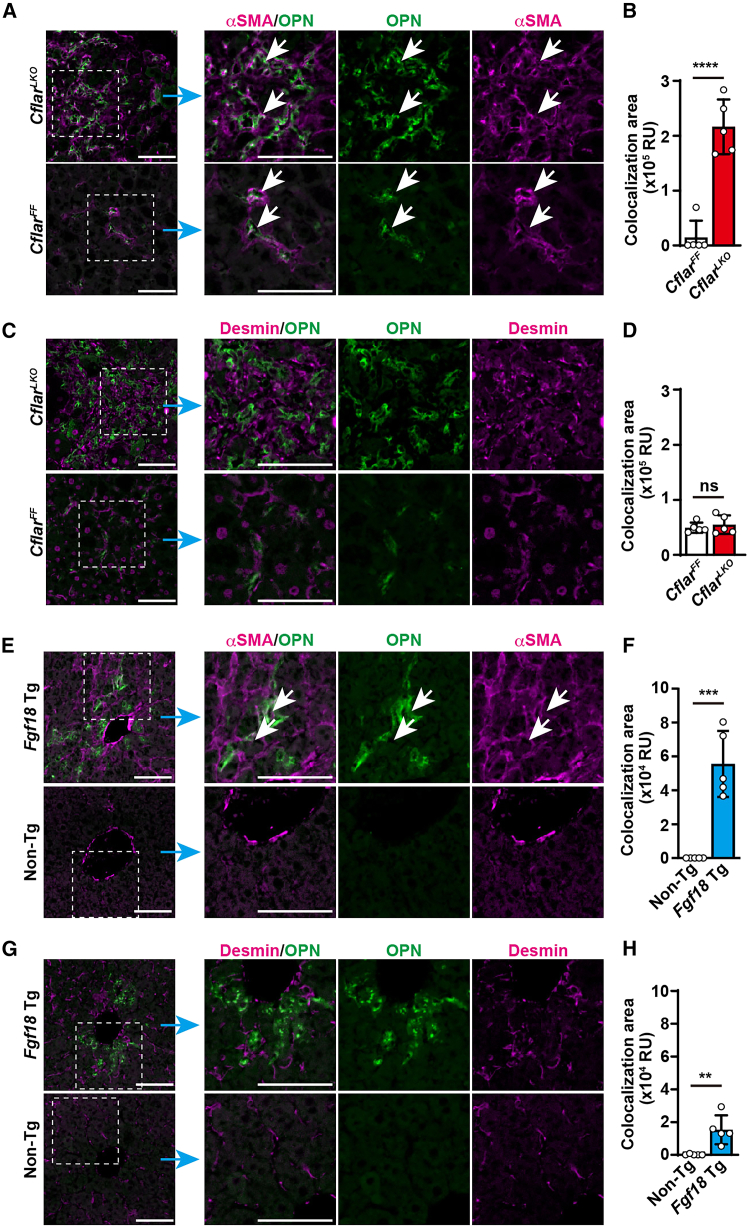
OPN is localized in αSMA^+^ activated HSCs/myofibroblasts but not desmin^+^ quiescent HSCs *in vivo* (A–D) Eight-week-old *Cflar*^*F/F*^ and *Cflar*^*LKO*^ mice (*n* = 5 mice per genotype) were fed the CDE diet for 4 weeks. Liver sections were stained with anti-OPN antibody (A and C) along with anti-αSMA (A) or anti-desmin antibodies (C). Merged signals are indicated by white arrows. Merged areas for both OPN and αSMA or OPN and desmin were quantified and are expressed as the relative area (B and D). Scale bars, 100 μm. (E–H), Eight-week-old non-Tg and *Fgf18* Tg mice (*n* = 5 mice per genotype) were sacrificed, and liver sections were stained and analyzed as described in (A and B). Statistical significance was determined by two-tailed unpaired Student’s *t* tests. ∗*p* < 0.05; ∗∗*p* < 0.01; ∗∗∗*p* < 0.001; ∗∗∗∗*p* < 0.0001; ns, not significant. The pooled results were obtained from two independent experiments.

We investigated the cellular sources of OPN expression to assess the *in vivo* relevance of our findings. Previous studies have reported that OPN is expressed in various cell types, including macrophages, hepatocytes, cholangiocytes, and T cells, depending on the experimental context.^30^ We performed immunostaining of liver tissues with antibodies against OPN, αSMA, and desmin. In *Cflar*^*LKO*^ mice fed a CDE diet for 4 weeks, the OPN^+^ areas partially overlapped with the αSMA^+^ regions but not with the desmin^+^ areas (Figures 4A–4D). A similar pattern was observed in the *Fgf18* transgenic mice (Figures 4E–4H). Notably, some OPN^+^ areas did not colocalize with αSMA^+^ staining, suggesting that other cell types, such as macrophages, T cells, and cholangiocytes also contribute to OPN production under these conditions. Collectively, these results indicate that αSMA^+^ cells are a major, but not exclusive, source of OPN *in vivo* in this liver fibrosis model *in vivo*.

### Myofibroblasts transmit signals to HSCs through an *Spp1/integrin*-dependent pathway

**Figure 5:**
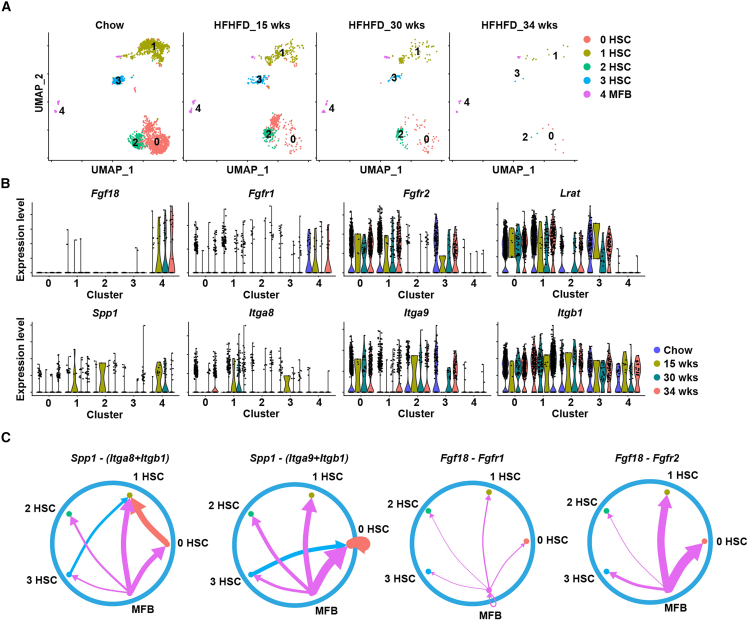
Myofibroblasts transmit signals to HSCs in an *Spp1*/*integrin*-dependent manner (A) UMAP plot showing the five clusters of HSCs and myofibroblasts from wild-type mice fed either a chow diet or an HFHFD for the indicated number of weeks (*n* = 3 mice per group). Each number indicates a cell cluster. (B) Violin plot showing the expression levels of the indicated genes in HSCs (clusters 0 to 3) and myofibroblasts (cluster 4) at the indicated time points. (C) Communication networks between HSCs and myofibroblasts, as analyzed using CellChat. The *Fgf18/Fgfr* and *Spp1/integrin receptor* pairs involved in signal transmission from myofibroblasts to HSCs are shown. The edge width represents the communication probability. MFB, myofibroblast; HSC, hepatic stellate cell. See also Figures S2 and S3.

We reanalyzed publicly available scRNA-seq data from the livers of mice fed a high-fat high-fructose diet (HFHFD) for 15, 30, or 34 weeks to evaluate the *in vivo* relevance of intercellular communication *in vivo*.^31^ Uniform manifold approximation and projection (UMAP) analysis of nonparenchymal liver cells revealed 19 distinct clusters (Figure S2). Among these clusters, we focused on those corresponding to HSCs and myofibroblasts. The UMAP analysis further subdivided these into four HSC clusters and one myofibroblast cluster (Figure 5A). Notably, the numbers of HSCs and myofibroblasts decreased over time, likely due to difficulties in recovering viable single cells from severely fibrotic tissues. The analysis of the violin plot showed that *Spp1* expression was enriched in myofibroblast cluster 4 at 15 and 30 weeks (Figure 5B). In the same cluster, *Fgf18* and *Fgfr1* were also highly expressed, whereas *Lrat* and *Fgfr2* were expressed primarily in HSC clusters (Figure 5B). Given that OPN signals via CD44 and several integrins,^32^ we employed CellChat^33^ to analyze ligand-receptor interactions. This analysis revealed that myofibroblast-derived *Spp1* signals to HSCs via multiple integrins (Figure 5C). Additionally, *Fgf18* secreted by myofibroblasts predominantly signaled to HSCs through *Fgfr2*, with weaker signaling through *Fgfr1*. These scRNA-seq data support our *in vitro* findings and indicate that myofibroblasts communicate with HSCs via both the *Fgf18*-*Fgfr1/2* and *Spp1*-*Integrin* signaling pathways, highlighting their involvement in the progression of liver fibrosis *in vivo*.

### *Spp1* mediates signals between different cancer-associated fibroblasts

We assessed whether OPN-mediated communication between HSCs and myofibroblasts extends beyond liver fibrosis by examining its relevance in cancer-associated fibroblasts (CAFs), a major cellular component of the tumor microenvironment.^34^^,^^35^ Previous studies have identified HSCs as a key source of CAFs in a murine intrahepatic cholangiocarcinoma model, where CAFs were further classified into inflammatory and growth factor-enriched CAFs (iCAFs), myofibroblastic CAFs (myCAFs), and mesothelial CAFs (mesCAFs).^36^ We reanalyzed a publicly available scRNA-seq dataset from that study. The UMAP analysis identified six distinct clusters, including iCAFs, myCAFs, and my/mesCAFs (Figure S3A). The UMAP and violin plots revealed that *Des*, *Fgfr2*, and *Lrat* were predominantly expressed in iCAFs, which presented low *Col1a2* expression (Figures S3B and S3C). In contrast, *Col1a2*, *Fgfr1*, and *Spp1* were highly expressed in myCAFs, whereas *Spp1* and *Fgfr1* were most enriched in mesCAFs. The cell-cell communication analyses further indicated that *Spp1* from my/mesCAFs signals to iCAFs via integrin receptors (Figure S3D). Together, these findings suggest that OPN-mediated intercellular communication is not restricted to *in vitro* settings or liver fibrosis, but may also occur between distinct CAF subsets in murine tumor models.

### FGF18 primarily targets HSCs, whereas FGF21 mainly induces signals in hepatocytes

**Figure 6:**
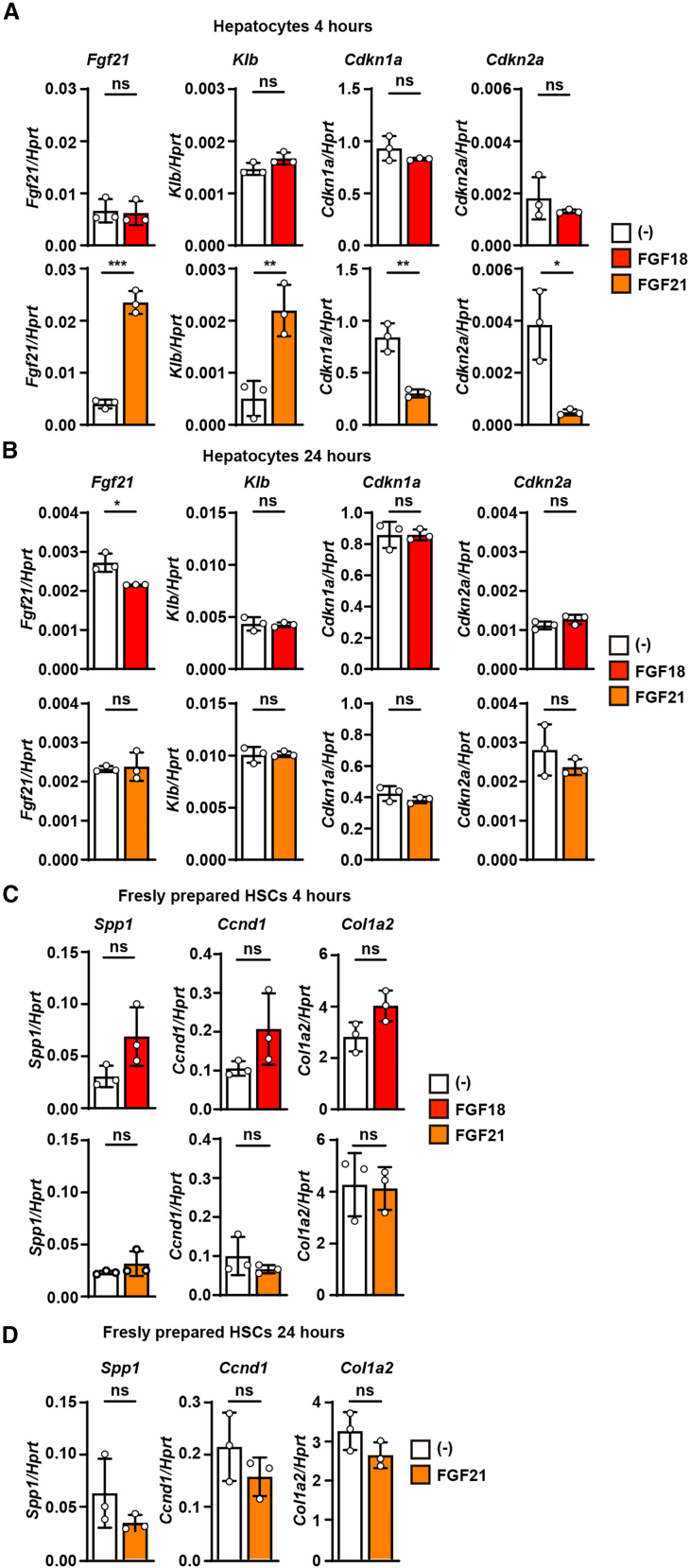
FGF18 primarily targets HSCs, whereas FGF21 mainly induces signals in hepatocytes (A and B) FGF21 increases the expression of *Fgf21* and *Klb* but downregulates the expression of senescence markers, including *Cdkn1a* and *Cdkn2a* in hepatocytes. Primary hepatocytes were isolated as in the STAR Methods and stimulated with FGF18 (100 ng/mL) or FGF21 (100 ng/mL) for 4 h (A) or 24 h (B). The expression of the indicated genes was analyzed via qPCR. Results are mean ± SD of triplicate samples. (C and D) FGF21 does not upregulate or downregulate the expression of profibrotic genes. HSCs were isolated as described in Figure 1A, serum-starved for 24 h, and then left untreated or stimulated with FGF18 (100 ng/mL) or FGF21 (100 ng/mL) for 4 h (C and D) or with FGF21 (100 ng/mL) for 24 h (D). The expression of the indicated genes was analyzed via qPCR. Results are expressed as the means ± SDs of triplicate samples. All results are representative of two independent experiments. Statistical significance was determined by two-tailed unpaired Student’s *t* tests. ∗*p* < 0.05; ∗∗*p* < 0.01; ∗∗∗*p* < 0.001; ∗∗∗∗*p* < 0.0001; ns, not significant.

Since FGF21 has been used to treat liver fibrosis by alleviating metabolic dysfunction,^17^^,^^37^ a comparison of its effects with those of FGF18 on HSCs and hepatocytes is of interest. While FGF18 failed to induce detectable signaling in primary hepatocytes, FGF21 upregulated *Fgf21* and *Klotho beta* (*Klb*) expression and concurrently suppressed the expression of the senescence markers *cyclin-dependent kinase inhibitor 1A* (*Cdkn1a*) and *Cdkn2a* at 4 hours poststimulation (Figures 6A and 6B). In contrast, FGF21 did not induce *Spp1* expression in HSCs cultured for 5 days at either 4 or 24 hours, whereas FGF18 robustly induced *Spp1* under the same conditions at 24 hours (Figures 2C, 6C, and 6D). These findings suggest that FGF21 primarily targets hepatocytes, whereas FGF18 acts on HSCs to promote proliferation and *Spp1* expression.

**Figure 7:**
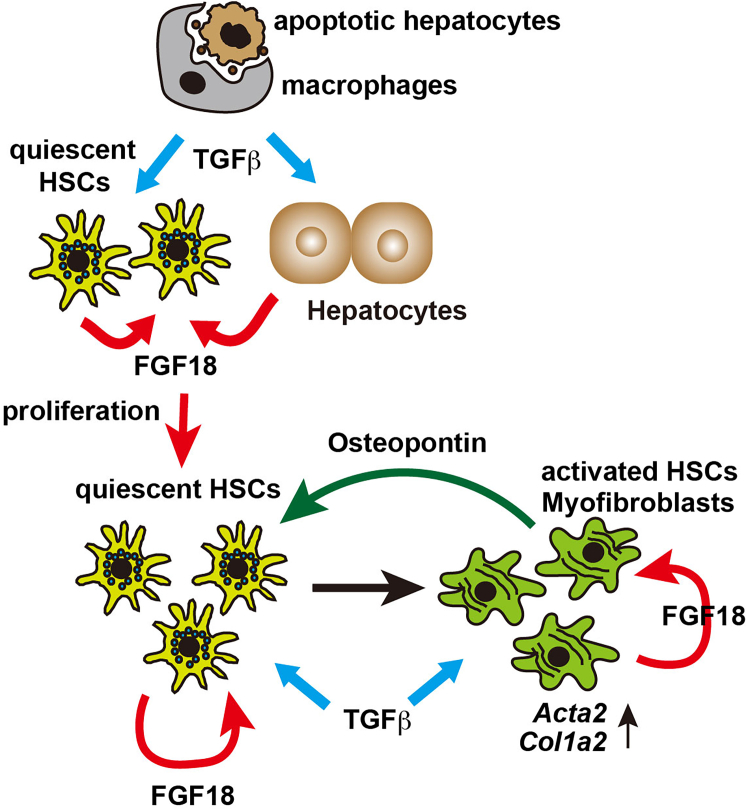
A proposed model how FGF18 promotes liver fibrosis in concert with TGFβ and OPN TGFβ produced by macrophages induces the expression of FGF18 in both HSCs and hepatocytes. FGF18, in turn, promotes the proliferation of quiescent HSCs. These proliferating HSCs are subsequently activated by profibrogenic stimuli such as TGFβ, leading to their transdifferentiation into αSMA^+^ myofibroblasts. In addition, FGF18 stimulates the production of OPN from activated HSCs/myofibroblasts. OPN acts in a paracrine manner to activate neighboring quiescent HSCs, thereby increasing the expression of profibrotic genes. This feedforward amplification loop is a key mechanism that drives the progression of liver fibrosis under specific pathological conditions.

Based on these data, we propose a model for how FGF18 promotes liver fibrosis in cooperation with TGFβ and OPN (Figure 7). TGFβ induces *Fgf18* expression in hepatocytes and HSCs and promotes HSC proliferation. Upon exposure to TGFβ or other cytokines, these proliferating HSCs become activated and differentiate into myofibroblasts. FGF18 further activates myofibroblasts, leading to OPN production. OPN then acts on quiescent HSCs, increasing the expression of profibrogenic genes. Through this mechanism, FGF18 establishes a positive feed-forward loop between quiescent HSCs and myofibroblasts via OPN signaling, thereby amplifying fibrogenic responses.

## Discussion

In this study, we identified *Spp1* as a novel downstream target of FGF18 signaling in HSCs. The induction of *Spp1* expression by FGF18 was strongly dependent on the activation status of HSCs, with significant upregulation observed in culture-activated αSMA^+^ HSCs (5-day-cultured HSCs) but not in freshly isolated quiescent HSCs. Notably, OPN secreted from FGF18-stimulated culture-activated HSCs was capable of activating quiescent HSCs and inducing profibrotic gene expression, thereby establishing an intercellular communication loop. This feed-forward interaction between FGF18 and OPN was further supported by *in vivo* data derived from murine liver fibrosis models.

Previous work from our group revealed that FGF18 promotes HSC proliferation while attenuating TGFβ-induced profibrotic gene expression,^19^ which is consistent with findings reported by others.^38^ Since FGF18 does not directly induce the expression of classical fibrotic genes, we performed bulk RNA-seq to identify alternative targets and found that *Spp1* was upregulated in FGF18-treated HSCs. Interestingly, although FGF18 and TGFβ exert opposing effects on gene programs, such as *Col1a2*/*Acta2* versus *Ccnd1*, they acted synergistically to increase *Spp1* expression in αSMA^+^ activated HSCs.

OPN is a multifunctional extracellular matrix protein involved in tissue remodeling, fibrosis, and cancer progression.^20^^,^^22^ Previous reports have shown that OPN is expressed by various cell types, including macrophages, CD4^+^ T cells, and cholangiocytes.^30^ We observed that FGF18 specifically induces OPN production in culture-activated αSMA^+^ HSCs and that OPN selectively promotes profibrotic gene expression in freshly prepared quiescent HSCs but not their activated counterparts, establishing a positive feedback loop between HSC subsets via FGF18-OPN signaling.

*In vivo* validation in murine models revealed that αSMA^+^ activated HSCs or myofibroblasts, but not desmin^+^ quiescent HSCs, expressed OPN in fibrotic livers. The results of the CellChat analyses of the scRNA-seq data supported this finding, as *Spp1*-mediated intercellular communication from myofibroblasts to HSCs via integrin pathways was identified.^31^ Similar *Spp1*-driven intercellular interactions have also been observed between my/mesCAFs and iCAFs in murine models of intrahepatic cholangiocarcinoma.^36^ Indeed, based on their gene expression profiles, myCAFs and iCAFs partially resemble myofibroblasts and quiescent HSCs, respectively; both myCAFs and myofibroblasts are characterized by the expression of *Acta2*, collagen-related genes, *Fgfr1*, and *Spp1*, while iCAFs and quiescent HSCs express *Des*, *Fgfr2*, and *Lrat*.^19^^,^^36^ Notably, *Spp1*/OPN^+^Col1a1^+^ CAFs have also been identified in castration-resistant prostate cancer, where the *Spp1*/OPN-ERK signaling axis has been implicated in driving therapeutic resistance.^39^ These findings suggest that *Spp1*/OPN-mediated cell-cell communication, potentially involving FGF18 signaling, are not restricted to fibrotic liver disease but may represent a broader mechanism of stromal crosstalk across multiple cancer types, including cholangiocarcinoma and prostate cancer. Of note, our present study did not indicate that FGF18 signaling contributes to OPN production in myofibroblasts *in vivo*. However, given that HSCs cultured for 5 days became αSMA^+^ cells and responded to FGF18 stimulation by upregulating *Spp1* expression, it is likely that FGF18 may play a role in OPN production in activated HSCs or myofibroblasts *in vivo*.

Mechanistically, siRNA-mediated knockdown experiments revealed distinct roles for FGFR isoforms in FGF18 signaling: FGFR2 primarily mediated *Ccnd1* induction, whereas FGFR1 selectively regulated *Spp1*expression. The scRNA-seq analyses further revealed differential expression patterns of *Fgfr1* and *Fgfr2* in myofibroblasts and HSCs, respectively. Inhibition of MEK/ERK signaling with U0126 suppressed both FGF18-induced *Ccnd1* expression and the synergistic induction of *Spp1* by FGF18 and TGFβ, indicating that MEK/ERK activity is essential for FGF18-mediated signaling. These results also suggest that TGFβ enhances FGF18 signaling through a noncanonical, non-SMAD pathway.

Additionally, FGF18-induced *Spp1* expression requires the preculture of HSCs on stiff substrates, implicating a role for mechanotransduction in this response. ECM stiffness is known to activate YAP/TAZ signaling,^40^ and fluid shear stress has been shown to increase *Spp1* expression, particularly in the absence of YAP1.^41^ Since FGF18 has been shown to reduce YAP1 protein levels,^38^ we speculate that FGF18-induced *Spp1* upregulation may be facilitated by YAP1 attenuation in combination with mechanical stress cues.

In contrast to FGF18, FGF21 is known to MASLD and MASH by improving glucose and lipid metabolism in hepatocytes.^42^^,^^43^ FGF21 agonists are currently under clinical evaluation for the treatment of MASH.^16^^,^^17^ Our findings suggest that FGF18 and FGF21 exhibit distinct cell type and signaling specificities, with FGF18 acting predominantly on HSCs and FGF21 targeting hepatocytes. This distinction may be partly due to differential expression of β-Klotho, a coreceptor required for FGF21 signaling.^44^ Indeed, FGF21 stimulation upregulated both *Fgf21* and *Klb* expression in hepatocytes, suggesting a feed-forward amplification loop. However, whether signaling specificity is fully dictated by β-Klotho remains to be determined.

Increased expression of FGF18 and OPN has been observed in fibrotic livers and various cancers.^19^^,^^20^^,^^38^^,^^45^^,^^46^ Our findings identify a feed-forward loop between these two signaling molecules. Previous work has demonstrated that targeting the neurotrophin-3/NTRK3 axis can suppress liver fibrosis by disrupting autocrine/paracrine HSC signaling.^10^ Notably, the hepatocyte-specific deletion of *Fgf18* only partially attenuated CDE-induced liver fibrosis in *Cflar*^*LKO*^ mice,^19^ suggesting that simultaneous blockade of FGF18 and OPN may be more effective. Future studies evaluating the therapeutic potential of the dual inhibition of FGF18 and OPN—both in liver fibrosis and in cancers with elevated *Fgf18* and *Spp1* expression—are warranted.

### Limitations of the study

This study has several limitations that warrant consideration. First, the number of HSCs that can be isolated from mice is limited, and these cells undergo spontaneous activation even under unstimulated conditions in standard culture systems. Consequently, we were unable to perform gene-editing experiments such as *CRISPR/Cas9*-mediated knockout to dissect FGF18-related signaling pathways in quiescent HSCs. Although immortalized HSCs retained partial responsiveness to FGF18, the magnitude of *Spp1* induction was substantially lower than that in freshly isolated cells. In relation to this issue, our current study did not fully clarify the molecular mechanisms by which FGF18 and TGFβ exert either synergistic or antagonistic effects in a gene-dependent manner. Therefore, future efforts to develop protocols for the large-scale isolation or *in vitro* expansion of quiescent HSCs are essential.

Second, although we identified a feed-forward regulatory loop between FGF18 and OPN *in vitro*, the physiological relevance of this interaction *in vivo* remains to be determined. Assessing whether interrupting this axis mitigates liver fibrosis in various murine models is important. In this context, treatment with a neutralizing antibody against FGF18 represents a promising strategy to evaluate the functional significance of FGF18 in *Spp1* expression and fibrogenesis. However, a major limitation is the lack of a high-affinity neutralizing antibody specific to murine FGF18. While we have successfully developed high-affinity antibodies targeting human FGF18,^47^ the generation of murine-specific antibodies is critical for *in vivo* validation. Addressing these limitations in future studies will further elucidate the role of the FGF18-OPN axis in liver fibrosis and may contribute to the development of novel antifibrotic therapies.

## Resource availability

### Lead contact

Further information and requests for resources and reagents should be directed to and will be fulfilled by the lead contact Hiroyasu Nakano (hiroyasu.nakano@med.toho-u.ac.jp).

### Materials availability

All the biological materials, including the hepatocyte-specific *Fgf18* Tg mice (B6.Cg-*Gt(ROSA)26Sor*^*tm(CAG-Fgf18)1N*^;B6.Cg-*Speer6-ps1*^*Tg(Alb-cre)21Mgn*^/J) used in this study, are available from the corresponding authors upon reasonable request. Obtaining *Cflar*^*FF*^ mice requires a material transfer agreement (MTA) with the organization described in the manuscript. In addition, patents are pending in Japan to use the hepatocyte-specific *Fgf18* Tg mice as an animal model of liver fibrosis.

### Data and code availability

The bulk RNA-seq datasets were deposited in NCBI under accession number GSE285364. Raw data from Figures 1, 2, 3, 4, 6, and S1 were deposited on Mendeley at https://doi.org/10.17632/cd72ysdp29.1. Other datasets generated and/or analyzed during the current study are available from the corresponding author upon reasonable request. All computer codes used in this study are available from the corresponding authors upon reasonable request.

## Acknowledgments

We thank Y.-W.He for providing *Cflar*^*FF*^ mice. This work was supported in part by Grants-in-Aid for Scientific Research (B) (20H03475 and 23H02707 to H.N.) and for Scientific Research (C) (17K08994 and 20K11589 to Y.T., and 25K10306 to T.S.) from the Japan Society for the Promotion of Science (JSPS); the Japan Agency for Medical Research and Development through AMED-CREST (23gm1210002 to M.T. and H.N.) from the Ministry of Education, Culture, Sports, Science, and Technology, Japan; the Princess Takamatsu Cancer Research Fund (to H.N.); the Takeda Science Foundation (to H.N.); and the Center for Diversity and Inclusion of Toho University’s Collaborative Research Enhancement Support (to T.S.).

## Author contributions

T.S. and H.N. designed the study and interpreted the results. T.S., S.K.-S., T.N., and Y.T. performed and analyzed most of the experiments. T.S. analyzed the scRNA-seq results. H.Y. provided critical reagents. T.M., K.O., and M.T. supervised the experiments. T.S. and H.N. wrote the manuscript with constructive input from all the authors.

## Declaration of interests

Y.T. and H.N. are inventors on the patent application that includes the generation of the transgenic mice that spontaneously developed liver fibrosis used in this study.

## STAR★Methods

### Key resources table

**Table:** 

REAGENT or RESOURCE	SOURCE	IDENTIFIER
**Antibodies**		

Anti-desmin rabbit monoclonal antibody	Abcam	Cat#AB32362; RRID: AB_731901
Anti-αSMA rabbit monoclonal antibody	Abcam	Cat#AB124964; RRID: AB_11129103
Anti-OPN goat polyclonal antibody	R&D	Cat#AF808; RRID: AB_2194992
Alexa 647-conjugated donkey anti-rabbit IgG	Invitrogen	Cat#A31573; RRID: AB_2536183
Alexa 594-conjugated donkey anti-goat IgG	Invitrogen	Cat#A32758; RRID: AB_2762828

**Bacterial and virus strains**		

pPS-EF1-SV40 large T antigen	This paper	N/A
pPS-EF1-LCS-T2A-RFP	System Bioscience	Cat#LF520A-1

**Chemicals, peptides, and recombinant proteins**		

Human FGF18	PeproTech	Cat#100-28
Human FGF21	PeproTech	Cat#100-42
Human TGFβ1	PeproTech	Cat#100-21
Mouse OPN	R&D	Cat#441-OP/CF
U0126 (MEK inhibitor)	Calbiochem	Cat#662005
Collagenase type IV	Sigma-Aldrich	Cat#C5138
DNase I	Sigma-Aldrich	Cat#DN25
Nycodenz	SEW	Cat#18003
Pronase E	KNF	Cat#KA-002
Fetal bovine serum	Gibco	Cat#10270106
High glucose DMEM	Nacalai Tesque	Cat#16971-55
Benzylpenicillin potassium	Meiji-seika-pharma	CAS: 113-98-4
Streptomycin sulfate	Meiji-seika-pharma	CAS: 3810-74-0
Normal donkey serum	Jackson ImmunoResearch	Cat#017-000-121
10%-formaldehyde neutral buffer solution	Nacalai Tesque	Cat#37152-51
Citrate buffer solution	LSI Medicine	Cat#RM-102C
Vectashield mounting medium with DAPI	Vector	Cat#H-1200
Blocking One Histo	Nacalai Tesque	Cat#06349-64
MaxBlock Autofluorescence Reducing Kit	MaxVision Biosciences	Cat#MB-L
Sepasol-RNA I SuperG	Nacalai Tesque	Cat#09379-55
RNAiMAX	Thermo Fisher Scientific	Cat#13778150
Opti-MEM	Thermo Fisher Scientific	Cat#31985070

**Critical commercial assays**		

Mouse/Rat Osteopontin DuoSet ELISA	R&D	Cat#DY441
RNeasy Micro Kit	QIAGEN	Cat#74004
ReverTra Ace qPCR RT Kit	Toyobo	Cat#FSQ-101
Fast SYBR™ Green Master Mix	Applied Biosystems	Cat#4385614
SMART-Seq HT PLUS Kit	TaKaRa Bio	Cat#R400748

**Deposited data**		

Bulk RNA-seq data	This paper	GEO: GSE285364
GSE188273	Tsuchiya et al.^19^	GEO: GSE188273
GSE99010	Tsuchida et al.^26^	GEO: GSE99010
GSE166504	Su et al.^30^	GEO: GSE166504
GSE154170	Affo et al.^34^	GEO: GSE154170
Raw data for Figures	This paper	https://doi.org/10.17632/cd72ysdp29.1

**Experimental models: Cell lines**		

Immortalized mouse HSCs (iHSCs)	This paper	N/A

**Experimental models: Organisms/strains**		

Mouse: *Cflar*^*F/F*^: B6.129-*Cflar*^*tm1Ywh*^/J	The Jackson Laboratory	JAX: 022009
Mouse: *Alb-Cre*: B6.Cg-*Speer6-ps1*^*Tg(Alb-cre)21Mgn*^/J	The Jackson Laboratory	JAX: 003574
Mouse: *Cflar*^*LKO*^: B6.129-*Cflar*^*tm1Ywh*^/J; B6.Cg-*Speer6-ps1*^*Tg(Alb-cre)21Mgn*^/J	Tsuchiya et al.^19^	N/A
Mouse: Non-Tg: B6.Cg-*Gt(ROSA)26Sor*^*tm(CAG-Fgf18)1N*^	Tsuchiya et al.^19^	N/A
Mouse: *Fgf18* Tg: B6.Cg-*Gt(ROSA)26Sor*^*tm(CAG-Fgf18)1N*^;B6.Cg-*Speer6-ps1*^*Tg(Alb-cre)21Mgn*^/J	Tsuchiya et al.^19^	N/A
Mouse: C57BL/6	Crea-Japan	N/A

**Oligonucleotides**		

*Acta2* Fwd primer: 5′-CTGACAGAGGCACCACTGAA-3′	This paper	N/A
*Acta2* Rev primer: 5′- CATCTCCAGAGTCCAGCACA-3′	This paper	N/A
*Col1a2* Fwd primer: 5′-CAGAACATCACCTACCACTGCAA-3′	This paper	N/A
*Col1a2* Rev primer: 5′-TTCAACATCGTTGGAACCCTG-3′	This paper	N/A
*Col3a1* Fwd primer: 5′-CTGTAACATGGAAACTGGGGAAA-3′	This paper	N/A
*Col3a1* Rev primer: 5′-CCATAGCTGAACTGAAAACCACC-3′	This paper	N/A
*Ccnd1* Fwd primer: 5′-CAGAAGTGCGAAGAGGAGGTC-3′	This paper	N/A
*Ccnd1* Rev primer: 5′-TCATCTTAGAGGCCACGAACAT-3′	This paper	N/A
*Spp1* Fwd primer: 5′-CACTCCAATCGTCCCTAC-3′	This paper	N/A
*Spp1* Rev primer: 5′-AGACTCACCGCTCTTCAT-3′	This paper	N/A
*Fgf18* Fwd primer: 5′-CCTGCACTTGCCTGTGTTTAC-3′	This paper	N/A
*Fgf18*: Rev primer: 5′-TGCTTCCGACTCACATCATCT-3′	This paper	N/A
*Fgfr1* Fwd primer: 5′-TAATACCACCGACAAGGAAATGG-3′	This paper	N/A
*Fgfr1* Rev primer: 5′-TGATGGGAGAGTCCGATAGAGT-3′	This paper	N/A
*Fgfr2* Fwd primer: 5′-AATCTCCCAACCAGAAGCGTA-3′	This paper	N/A
*Fgfr2* Rev primer: 5′-CTCCCCAATAAGCACTGTCCT-3′	This paper	N/A
*Fgf21* Fwd primer: 5′-CCTTGAAGCCAGGGGTCATT-3′	This paper	N/A
*Fgf21* Rev primer: 5′-AGGATCAAAGTGAGGCGATCC-3′	This paper	N/A
*Klb* Fwd primer: 5′-GACACAACCTGATCAAGGCAC-3′	This paper	N/A
*Klb* Rev primer: 5′-CCTTCTGATGAGGGCGGAAG-3′	This paper	N/A
*Cdkn1a* Fwd primer: 5′-ATCCAGACATTCAGAGCCACAG-3′	This paper	N/A
*Cdkn1a* Rev primer: 5′-CAAAGTTCCACCGTTCTCGG-3′	This paper	N/A
*Cdkn2a* Fwd primer: 5′-TGGTCACTGTGAGGATTCAGC-3′	This paper	N/A
*Cdkn2a* Rev primer: 5′-TTGCCCATCATCATCACCTGG-3′	This paper	N/A
*Hprt* Fwd primer: 5′-AACAAAGTCTGGCCTGTATCCAA-3′	This paper	N/A
*Hprt* Rev primer: 5′-GCAGTACAGCCCCAAAATGG-3′	This paper	N/A
siRNA targeting mouse *Fgfr1*	Dharmacon	Cat#L-040832-00-0005
siRNA targeting mouse *Fgfr2*	Dharmacon	Cat#L-040288-00-0005
Nontargeting siRNA	Dharmacon	Cat#D-001810-10-05

**Software and algorithms**		

RaNA-seq	Prieto et al.^48^	https://ranaseq.eu
GraphPad Prism 9 and 10	GraphPad Software	https://www.graphpad.com/
ZEN software	Zeiss	https://www.zeiss.com/microscopy/int/products/microscope-software/zen.html
QuantStudio Design & Analysis Software v2.6	Thermo Fisher Scientific	https://www.thermofisher.com/
R (version 4.3.0)	R Foundation	https://www.r-project.org/
BiocManager (version 1.30.25)	Bioconductor	https://www.bioconductor.org/
CellChat (version 1.6.1)	Jin et al.^32^	https://github.com/sqjin/CellChat
ImageJ	NIH	https://imagej.nih.gov/ij/
Fiji	NIH	https://imagej.net/software/fiji/downloads

**Other**		

QuantStudio 3 Real-Time PCR System	Thermo Fisher Scientific	Cat#A28137
Poly-L-lysine-coated 12 mm microcoverslips	MATSUNAMI	Cat#C012001
Collagen I-coated 6-well plates	Iwaki Glass	Cat#4810-010
LSM 880 confocal microscope	Zeiss	LSM880
NovaSeq 6000 system	Illumina	N/A
Agilent 2100 Bioanalyzer	Agilent Technologies	G2939BA
All-in-One microscope (BZ-X700)	KEYENCE	Model#BZ-X700

### Experimental model and study participant details

*Albumin-Cre* transgenic mice (*Alb-Cre*; stock #003574, The Jackson Laboratory) and C57BL/6 mice (Crea-Japan) were used in this study. *Cflar*^*flox/flox*^ (*Cflar*^*F/F*^) mice^49^ (provided by Dr. Y. H. He) were crossed with *Alb-Cre* mice to generate hepatocyte-specific *Cflar* knockout (*Cflar*^*LKO*^) mice.^19^ *Rosa26-LSL-Fgf18* transgenic (non-Tg) mice were crossed with *Alb-Cre* mice, yielding *Fgf18* transgenic (*Fgf18* Tg) mice with liver-specific *Fgf18* expression.^19^ Both male and female *Fgf18* Tg mice were used for the analysis. Liver fibrosis was induced by feeding 8-week-old female *Cflar*^*F/F*^ and *Cflar*^*LKO*^ mice a choline-deficient, ethionine-supplemented (CDE) diet for 4 weeks. All mice were maintained under specific pathogen-free (SPF) conditions at 23 ± 2°C and 55% ± 5% humidity on a 12-hour light/dark cycle. All animal procedures were conducted in accordance with the institutional guidelines approved by the Animal Experiment Committee of Faculty of Medicine, Toho University (protocol numbers 23-551 and 23-554).

### Method details

#### Isolation of HSCs from wild-type mice

HSCs were isolated from wild-type female C57BL/6 mice (>24 weeks old) using the previously described Nycodenz density gradient centrifugation method.^28^ Aging increases the number of vitamin A-containing lipid droplets in HSCs, facilitating their enrichment for downstream analyses.^50^ Briefly, the livers were first perfused via the portal vein with 0.3 mg/mL collagenase for 8 minutes, followed by a second perfusion with liver perfusion buffer 2 (LPB2: 136 mM NaCl, 5.4 mM KCl, 5 mM CaCl_2_, 0.5 mM NaH_2_PO_4_·2H_2_O, 0.42 mM Na_2_HPO_4_, 10 mM HEPES pH 7.5, 5 mM glucose, and 4.2 mM NaHCO_3_) containing 0.6 mg/mL pronase E (KA-002, KNF) and 0.06 mg/mL DNase I at a flow rate of 3 mL/min for 8 minutes. The perfused livers were transferred to a glass dish and manually dissociated in LPB2 containing 0.6 mg/mL pronase and collagenase. The cell suspension was incubated at 37°C for 10 minutes with gentle stirring, filtered through a 70 μm strainer, and centrifuged at 600 × *g* for 10 minutes. The resulting pellet was resuspended in GBSS/B buffer (136 mM NaCl, 5 mM KCl, 1 mM MgCl_2_·6H_2_O, 0.28 mM MgSO_4_·7H_2_O, 0.22 mM KH_2_PO_4_, 0.42 mM Na_2_HPO_4_·12H_2_O, 5.5 mM glucose, 2.7 mM NaHCO_3_, and 1.5 mM CaCl_2_·2H_2_O) containing 0.06 mg/mL DNase I and centrifuged twice at 600 × *g* for 10 minutes. The cells were then resuspended in GBSS/B supplemented with 8.8% Nycodenz (18003, SEW), and additional GBSS/B was layered gently on top. Following centrifugation at 1,500 × *g* for 22 minutes without braking, HSCs were collected from the white interphase band using a Pasteur pipette and washed by centrifugation at 600 × *g* for 10 minutes. Typically, ∼1.0 × 10^6^ HSCs were obtained from two mice, with 80–90% purity confirmed by UV autofluorescence under a confocal microscope.

#### Cell culture

Freshly prepared HSCs were plated at a density of 1 × 10^5^ cells per well in 48-well plates and serum-starved in DMEM containing 0.2% FBS for the indicated durations. Cells were then stimulated for 24 hours with FGF18 (100 ng/mL), TGFβ1 (1 ng/mL), OPN (100 ng/mL), or a combination of FGF18 (100 ng/mL) and TGFβ1 (1 ng/mL) in the presence or absence of the MEK inhibitor U0126 (10 μM). Freshly isolated HSCs were immortalized with a lentiviral vector encoding SV40 large T antigen (pPS-EF1-SV40 T Ag). This vector was generated by subcloning a PCR-amplified SV40 large T antigen fragment, obtained from HEK293T cells, into the pPS-EF1-LCS-T2A-RFP vector (System Biosciences, LF520A-1). Immortalized HSCs were cultured in DMEM supplemented with 10% FBS.

#### Bulk RNA-seq analysis of HSCs

Total RNA was extracted from HSCs using an RNeasy Micro Kit (74004, QIAGEN). The RNA libraries were prepared with the SMART-Seq HT PLUS Kit (R400748, TaKaRa Bio) according to the manufacturer’s protocol. Briefly, first-strand cDNA was synthesized by SMARTScribe reverse transcriptase with template switching activity at the 5′ end of the RNA template. After PCR amplification and purification of the double-stranded cDNA, the libraries were enzymatically fragmented and ligated with stem‒loop adapters. The libraries were then amplified and indexed with unique dual indices. Library quality and quantity were assessed using an Agilent 2100 Bioanalyzer with a High Sensitivity DNA Kit (Agilent Technologies). Paired-end sequencing (2 × 150 bp) was performed on a NovaSeq 6000 system (Illumina) to generate approximately 40 M reads (20 M pairs) per sample, with a target of 6G bases per sample. The sequences were subsequently mapped to the mouse reference genome GRCm38. RNA-seq data were normalized and analyzed for differential gene expression using the online platform RaNA-seq (https://ranaseq.eu).^48^ The RNA-seq data were deposited in NCBI under the GEO accession number GSE285364.

#### Correlation analysis of the expression of *Spp1* and other profibrotic genes

Relative expression levels of the indicated genes were retrieved from the bulk RNA-seq datasets GSE188273 ^19^ and GSE99010.^26^ GSE188273 includes liver samples from 12-week-old *Cflar*^*F/F*^ and *Cflar*^*LKO*^ mice fed a CDE diet for 4 weeks, as well as from 8-week-old non-transgenic (non-Tg) and *Fgf18* transgenic (*Fgf18* Tg) mice. GSE99010 comprises datasets from wild-type mice injected with CCl_4_ and fed either a normal diet (ND) or a Western diet (WD) for 12 or 24 weeks. Pearson’s correlation coefficients and two-tailed *p*-values were calculated.

#### Immunofluorescence analyses

Isolated HSCs were seeded onto poly-L-lysine-coated 12 mm microcoverslips (C012001, MATSUNAMI) placed in 24-well plates and cultured for the indicated time periods. After non-adherent cells were removed, the adherent cells were fixed with 4% paraformaldehyde, permeabilized with 0.1% Triton X-100, and stained with anti-αSMA or anti-desmin antibodies, followed by incubation with Alexa Fluor 647-conjugated donkey anti-rabbit secondary antibody. αSMA^+^ and desmin^+^ cells were manually counted and expressed as a percentage of the total number of DAPI^+^ total cells. Microscopy images were acquired using a BZ-X700 All-in-One fluorescence microscope (KEYENCE), and total cell numbers were automatically quantified using the hybrid cell counting function of the BZ-X700 system.

#### Quantitative polymerase chain reaction (qPCR) assays

Total RNA was extracted from HSCs using the RNeasy Micro Kit (74004, QIAGEN) and from hepatocytes using Sepasol-RNA I SuperG (09379-55, Nacalai Tesque), following the manufacturers’ protocols. Complementary DNA (cDNA) was synthesized using the ReverTra Ace qPCR RT Kit (FSQ-101, Toyobo). Quantitative PCR was conducted on a QuantStudio 3 Real-Time PCR System (Thermo Fisher Scientific) using SYBR Green chemistry. Gene expression levels were normalized to the endogenous control *Hprt*, and analyzed using QuantStudio Design & Analysis Software v2.6. The sequences of the primers used are listed in the STAR Methods section.

#### Enzyme-linked immunosorbent assay (ELISA)

OPN levels in HSC culture supernatants were measured using the Mouse/Rat Osteopontin DuoSet ELISA Kit (DY441, R&D Systems) according to the manufacturer’s protocol. Briefly, ELISA plates were coated with capture anti-OPN antibody diluted in 0.5% BSA–PBS and incubated overnight at 4°C. After blocking with 1% BSA–PBS containing 0.05% Tween-20 (PBS-T) for 1 hour at room temperature (RT), the plates were washed three times with PBS-T. Culture supernatants were then added and incubated for 1 hour at RT. After washing, biotinylated detection anti-OPN antibody was added and the plates were incubated for 2 hours at RT, followed by incubation with HRP-conjugated streptavidin incubation for 20 minutes. Signal development was performed using the TMB substrate, according to standard procedures. Protein levels were normalized by setting the average protein concentration of untreated supernatants to 1.0. The concentrations of OPN in the culture supernatants were then normalized by dividing the measured OPN levels by the corresponding relative protein concentrations.

#### siRNA transfection

For siRNA-mediated knockdown experiments, freshly prepared HSCs (1 × 10^5^ cells/well) were serum-starved and then transfected with siRNAs against nontargeting controls (D-001810-10-05, Dharmacon), *Fgfr1* (L-040832-00-0005, Dharmacon), *Fgfr2* (L-040288-00-0005, Dharmacon), or a combination of *Fgfr1* and *Fgfr2* using Lipofectamine RNAiMAX (13778150, Thermo Fisher Scientific) according to the manufacturer’s protocol. Reverse transfection was initially performed, followed by a second (forward) transfection 17 hours later. For both steps, siRNAs and RNAiMAX were diluted in Opti-MEM (31985070, Thermo Fisher Scientific). At 17 hours after the second transfection, culture media were replaced with fresh media, and cells were either left untreated or stimulated with FGF18 (100 ng/mL) for an additional 24 hours.

#### Histological analyses

For histological analysis, paraffin-embedded liver sections (3 to 4 μm thick) were pretreated with the MaxBlock™ Autofluorescence Reducing Kit (MaxVision Biosciences) according to the manufacturer’s instructions. Antigen retrieval was subsequently performed using Instant Citrate Buffer Solution (RM-102C, LSI Medicine). The sections were then blocked with Blocking One Histo (06349-64, Nacalai Tesque) supplemented with 5% donkey serum. Immunofluorescence staining was performed using primary antibodies against αSMA, desmin, or osteopontin (OPN), followed by incubation with Alexa Fluor-conjugated secondary antibodies. Imaging was performed using a Zeiss LSM 880 confocal microscope, and image acquisition and processing were conducted with ZEN software (Zeiss). The quantification of αSMA^+^, desmin^+^, and OPN^+^ areas was performed using the Stack Colocalization Analyzer plugin in Fiji (ImageJ, NIH), with uniform threshold settings applied across all images.

#### Reanalyses of the extracted scRNA-seq datasets

Previously published single-cell RNA sequencing (scRNA-seq) datasets (GSE166504 and GSE154170) were reanalyzed using R (v4.3.0) and BiocManager (v1.30.25). For the GSE166504 dataset,^31^ cells were filtered based on standard quality control criteria: nFeature_RNA > 300, nCount_RNA < 60,000, and mitochondrial gene percentage < 80%. The data were log-normalized and 2,000 variable features were identified. Principal component analysis (PCA) was performed using 30 dimensions, followed by UMAP for dimensionality reduction and clustering (resolution = 0.2) based on the top 30 principal components. HSCs and myofibroblasts were identified using canonical markers (HSCs: *Lrat* and *Reln*; myofibroblasts: *Acta2* and *Msln*) and subjected to subclustering (resolution = 0.1) using the same workflow. For the GSE154170 dataset,^36^ a Seurat object was created using a minimum threshold of 3 cells per gene and 200 genes per cell. Quality control filtering was applied (nFeature_RNA between 200 and 4,000; mitochondrial gene percentage < 10%). The data were normalized with a scale factor of 10,000, and 2,000 variable features were selected using the “vst” method. PCA and JackStraw analyses were performed to determine significant dimensions. UMAP visualization and clustering were performed using the top 4 principal components (resolution = 0.1). The cell-type annotation was based on marker information from the original publication. Intercellular communication networks were inferred using CellChat (v1.6.1). For the GSE166504 dataset,^31^ cluster labels were incremented by one for consistency. Analyses were performed using the built-in CellChat mouse ligand–receptor database. Overexpressed genes and interactions were identified with a p-value threshold of 0.5. The communication probabilities were computed using a truncated mean (trim = 0.05) with population size correction. Interactions were filtered to retain those present in at least one cell and aggregated using a threshold of 0.01. For the GSE154170 dataset,^36^ fibroblasts were categorized into CAF subtypes (iCAFs, myCAFs, mesCAFs, my/mesCAFs, and other CAFs). The communication analysis was conducted using the default parameters, and only interactions present in ≥10 cells were retained. Signaling pathways involving *Spp1* and *Fgf18* were visualized using the built-in functions of CellChat’s, including circle plots depicting the number and strength of interactions across cell populations. UMAP and feature plots were generated using Seurat visualization tools. The expression patterns of key genes (*Fgf18*, *Spp1*, *Fgfr1*, and *Fgfr2*) were visualized using gray-to-red feature plots and violin plots (point size = 0.1). The cell-cell communication outputs were visualized via CellChat using circle plots, aggregated network views, and contribution plots.

#### Isolation of hepatocytes

Hepatocytes were isolated using a modified two-step collagenase perfusion method as previously described.^19^ Briefly, livers from 8-week-old C57BL/6 mice were perfused via the portal vein with liver perfusion buffer 1 (LPB1: 136 mM NaCl, 5.4 mM KCl, 0.5 mM EGTA, 0.5 mM NaH_2_PO_4_·2H_2_O, 0.42 mM Na_2_HPO_4_, 10 mM HEPES pH 7.5, 5 mM glucose, and 4.2 mM NaHCO_3_) at a flow rate of 3 mL/min for 5 minutes. This step was followed by perfusion with liver perfusion buffer 2 (LPB2) containing 0.5 mg/mL collagenase type IV (C5138, Sigma‒Aldrich) and 0.06 mg/mL DNase I (DN25, Sigma‒Aldrich) for 8 minutes at the same flow rate. The digested livers were transferred to a sterile glass dishes, and connective tissues were carefully removed using forceps. The cell suspension was filtered, resuspended in DMEM, and centrifuged at 50 × *g* for 2 minutes to pellet the hepatocytes. The hepatocyte pellet was washed and resuspended in DMEM supplemented with 10% FCS, and plated onto collagen I-coated 6-well plates (4810-010, Iwaki Glass). After 24 hours, the medium was replaced to remove unattached, non-viable cells. For stimulation experiments, primary hepatocytes (4 × 10^5^ cells/well) were treated with FGF18 (100 ng/mL) or FGF21 (100 ng/mL) for 4 or 24 hours.

### Quantification and statistical analysis

Statistical analyses were performed by Pearson correlation coefficient analyses, two-tailed unpaired Student’s *t* test, two-way ANOVA with Sidak’s multiple comparisons test, two-way ANOVA with Tukey’s multiple comparisons test, one-way ANOVA with Dunnett’s multiple comparisons test, or one-way ANOVA with Tukey’s multiple comparisons test using GraphPad Prism 9 and 10. *p* value <0.05 was considered significant.

## Supplemental information

Document S1. Figures S1–S3

Table S1. Upregulated genes in HSCs treated with FGF18 or TGFβ, related to Figure 1
