# Supplementary material for: Intercellular communication between hepatic stellate cells and myofibroblasts mediated by osteopontin and FGF18 promotes liver fibrosis
Source: iScience. 2025 Jun 17;28(7):112932. doi: 10.1016/j.isci.2025.112932 (PMC12269570; doi:10.1016/j.isci.2025.112932)
Supplement: Document S1. Figures S1–S3 [file mmc1.pdf]

iScience, Volume *28*

Supplemental information

Intercellular communication between hepatic stellate cells and myofibroblasts mediated by osteopontin and FGF18 promotes liver fibrosis

Takao Seki, Sachiko Komazawa-Sakon, Takashi Nishina, Tetuo Mikami, Hideo Yagita, Katsuhide Okunishi, Minoru Tanaka, Yuichi Tsuchiya, and Hiroyasu Nakano

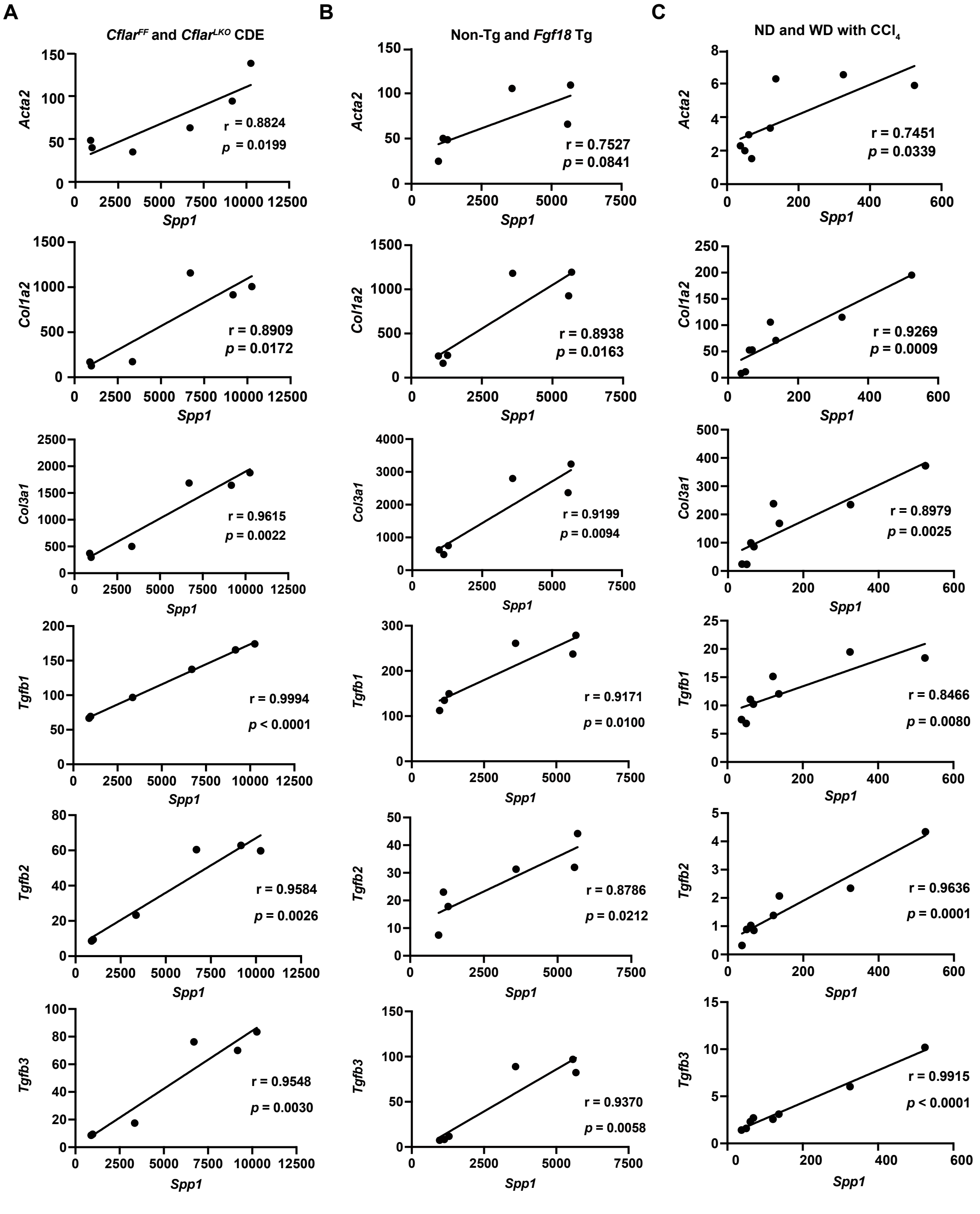

Figure S1. Correlation between the expression of *Spp1* and profibrotic genes, Related to Figure 1.

(A and B) Bulk RNA-seq datasets (GSE188273) from the livers of 12-week-old *Cflar*$^{F/F}$ and *CflarLKO* mice fed a CDE diet for 4 weeks (*n* = 3 mice per group) (A), and 8-week-old non-Tg and *Fgf18* Tg mice (*n* = 3 mice per group) (B), were retrieved and

reanalyzed.

(C) Bulk RNA-seq datasets (GSE99010) from the livers of wild-type mice injected with CCl_4 and fed either a normal diet (ND) or a Western diet (WD) for 12 or 24 weeks (pooled RNA from $n = 4$ mice per condition) were retrieved and reanalyzed. The correlations between the expression of *Spp1* and the indicated genes was assessed by Pearson correlation coefficient analysis. *P* values were calculated using a two-sided test.

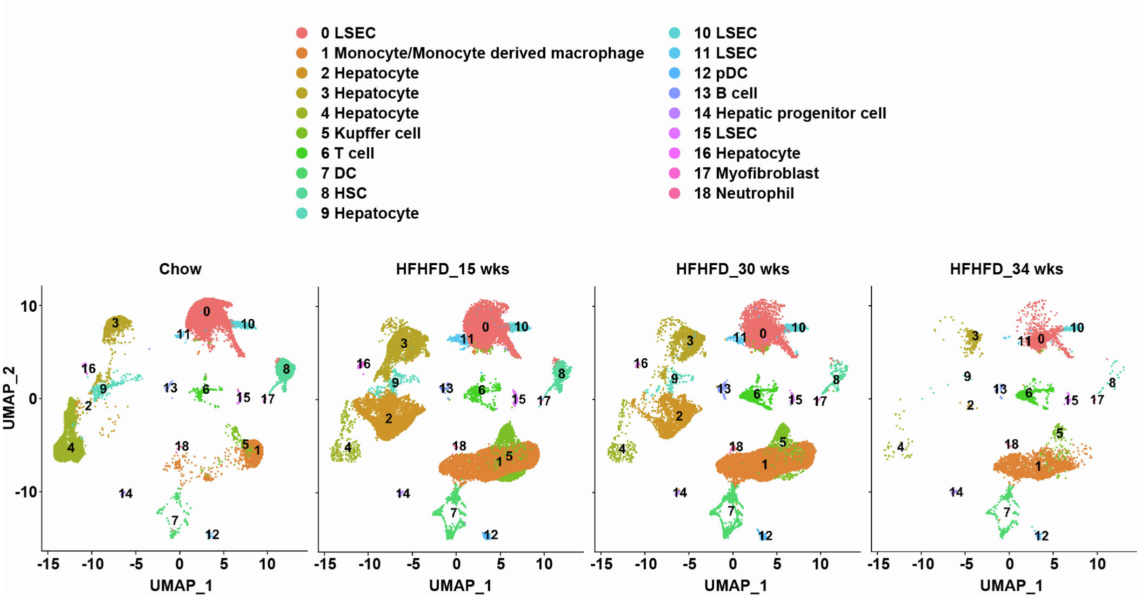

Figure S2. **Single-cell analyses of liver cell populations in wild-type mice subjected to different dietary conditions, Related to Figure 5.**

Liver single-cell transcriptomic data (GSE166504) from wild-type mice fed either a chow diet or a high-fat, high-fructose diet (HFHFD) for 15, 30, or 34 weeks were retrieved and reanalyzed using UMAP. A total of 19 distinct cell clusters are shown.

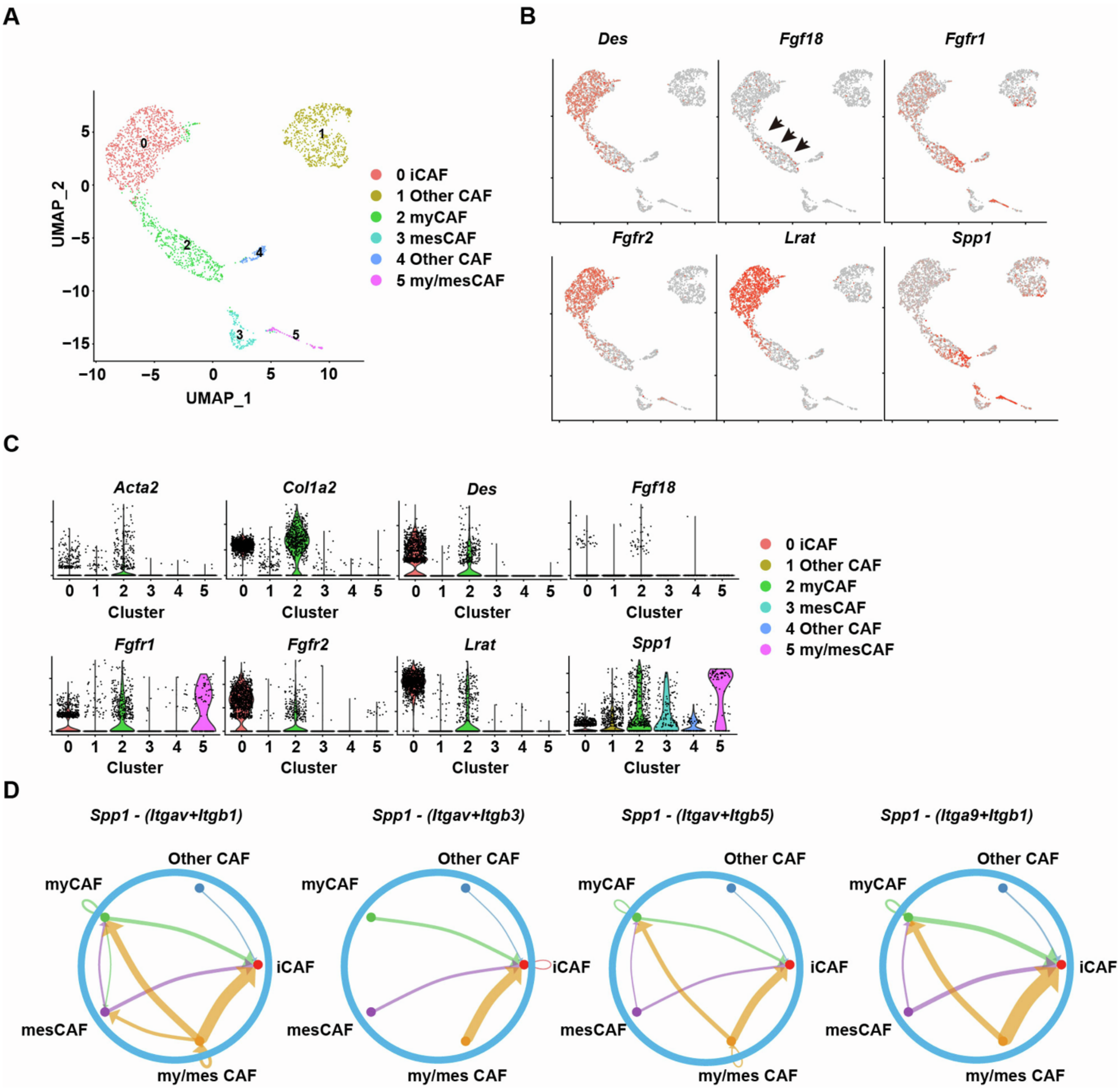

Figure S3. ***Spp1*** **mediates signaling between different CAFs, Related to Figure 5.**

(A) UMAP plot showing six distinct clusters of cancer-associated fibroblasts (CAFs) from the liver of a mouse with intrahepatic cholangiocarcinoma (n = 1). Each number represents an individual CAF cluster.

(B) Feature plots showing the expression of the indicated genes across the identified CAF clusters. The black arrows indicate *Fgf18*-expressing cells.

(C) Violin plots illustrating the expression levels of the indicated genes in each CAF subtype.

(D) Cell–cell communication network among CAF populations, as inferred by CellChat

analysis. *Spp1*/integrin receptor pairs that mediate inter-CAF signaling are shown. The edge width reflects the communication probability.

Abbreviations: iCAF, inflammatory and growth factor-enriched CAF; myCAF, myofibroblastic CAF; mesCAF, mesothelial CAF; my/mesCAF, hybrid myofibroblastic/mesothelial CAF.
